# Soluble Urokinase-Type Plasminogen Activator Receptor Levels as a Predictor of Kidney Replacement Therapy in Septic Patients with Acute Kidney Injury: An Observational Study

**DOI:** 10.3390/jcm11061717

**Published:** 2022-03-19

**Authors:** Tomasz Skalec, Barbara Adamik, Katarzyna Kobylinska, Waldemar Gozdzik

**Affiliations:** 1 Clinical Department of Anaesthesiology and Intensive Therapy, Wroclaw Medical University, Borowska St. 213, 50-556 Wroclaw, Poland; tomasz.skalec@umw.edu.pl (T.S.); waldemar.gozdzik@umw.edu.pl (W.G.); 2 Faculty of Mathematics, Informatics and Mechanics, University of Warsaw, Banacha 2, 02-097 Warsaw, Poland; kz277838@students.mimuw.edu.pl

**Keywords:** biomarkers, sepsis, septic shock, acute kidney injury, renal replacement therapy, kidney replacement therapy

## Abstract

The soluble urokinase-type plasminogen activator receptor (suPAR) is involved in the pathogenesis of acute kidney injury (AKI). Our goal was to establish the optimal suPAR cut-off point for predicting the need for kidney replacement therapy (KRT) use in sepsis patients and to analyze survival rates based on the suPAR level, AKI diagnosis, and the requirement for KRT. In total, 51 septic patients were included (82% septic shock; 96% mechanically ventilated, 35% KRT). Patients were stratified according to the AKI diagnosis and the need for KRT into three groups: AKI(+)/KRT(+), AKI(+)/KRT(−), and AKI(−)/KRT(−). A control group (*N* = 20) without sepsis and kidney failure was included. Sepsis patients had higher levels of the suPAR than control (13.01 vs. 4.05 ng/mL, *p* < 0.001). On ICU admission, the suPAR level was significantly higher in the AKI(+)/KRT(+) group than in the AKI(+)/KRT(−) and AKI(−)/KRT(−) groups (18.5 vs. 10.6 and 9.5 ng/mL, respectively; *p* = 0.001). The optimal suPAR cut-off point for predicting the need for KRT was established at 10.422 ng/mL (area under the curve 0.801, sensitivity 0.889, specificity 0.636). Moreover, patients AKI(+)/KRT(+) had the lowest probability of survival compared to patients AKI(+)/KRT(−) and AKI(−)/KRT(−) (*p* = 0.0003). The results indicate that the suPAR measurements may constitute an important element in the diagnosis of a patient with sepsis.

## 1. Introduction

The urokinase-type plasminogen activator receptor (uPAR) is a signaling glycoprotein with pleiotropic biological effects [1]. This protein is expressed in many cell types including immunologically active cells, endothelial cells, and podocytes [2]. Both the membrane-bound form of the receptor (uPAR) and the soluble form (suPAR), which is produced by the cleavage of the membrane-bound urokinase-type plasminogen activator receptor, can be detected in blood, urine, and other body fluids [3,4]. Numerous studies have shown that elevated levels of the suPAR are associated with inflammation and infection in a variety of acute diseases including sepsis, burn injuries, and meningitis [5,6,7].

Previously published data suggest that the suPAR plays a role in the pathogenesis of acute and chronic kidney disease, such as focal segmental glomerulosclerosis or diabetic nephropathy, and it can be used as a predictive marker of chronic kidney disease [8,9,10]. In patients with cardiovascular disease, increased levels of the suPAR in blood were associated with a diagnosis of chronic kidney disease and a decrease in the estimated glomerular filtration rate (eGFR) [11]. Findings based on an animal model suggest that kidney disease only develops when the suPAR activates some level of the podocyte β_3_ integrin, and it has been hypothesized that the suPAR itself causes kidney disease by damaging podocytes [12]. In a recently published study, it was shown that the suPAR might be directly involved in the pathogenesis of acute kidney injury (AKI) in humans by sensitizing kidney proximal tubules to damage by modulating cellular energy production and increasing oxidative stress [13].

Sepsis is the most common cause of AKI in critically ill patients and is often associated with a need for kidney replacement therapy (KRT) [14]. The goals of KRT are to replace excretory kidney function and to allow functional recovery of the kidneys. There has been a longstanding dilemma on when to start KRT in the case of severe AKI. While underdosing of KRT increases sepsis mortality, increasing the dose of KRT above the required level does not improve survival [15]. Currently, clinical judgment and two functional biomarkers, serum creatinine, and urine output are used to define AKI. Changes in creatinine concentration following kidney injury are delayed and not always representative of true kidney damage and are, therefore, ineffective in predicting outcome in AKI patients. In recent years, many efforts have been made to improve the early diagnosis of AKI, including the discovery and validation of new AKI biomarkers [16,17,18,19]. Based on the recently published recommendations for AKI biomarkers, current evidence from clinical trials supports the use of new biomarkers in the treatment of AKI. However, there are still large gaps in the knowledge that require further studies [20]. The involvement of the suPAR in the pathogenesis of AKI indicates that this protein may be useful as a biomarker for predicting treatment outcomes in septic patients with AKI, but there are few published studies assessing the diagnostic accuracy of suPAR levels in predicting the need for kidney replacement therapy. Nusshag et al. showed in a recently published study that the suPAR, together with the tissue inhibitor of metalloproteinase-2 and insulin-like growth factor-binding protein 7, were of diagnostic value for predicting septic acute kidney injury courses requiring KRT [21].

This study examined the relationship between blood suPAR levels and the need for KRT in AKI patients meeting Sepsis-3 criteria. Our goal was to establish the optimal suPAR cut-off point for predicting the need for KRT in sepsis patients and to analyze survival rates based on the suPAR level, AKI diagnosis, and the requirement for KRT. The suPAR level was hypothesized to be higher in AKI patients, especially those who required KRT to support kidney function.

## 2. Materials and Methods

### 2.1. Study Design

This single-center, prospective observational study was conducted at the Department of Anesthesiology and Intensive Therapy at the tertiary care University Hospital between March and December 2016. The study protocol complies with the 1975 Declaration of Helsinki as revised in 1983. The study protocol was approved by the local Bioethical Committee of Wroclaw Medical University (KB–58/2016) and informed consent was obtained from the patient or the patient’s representative.

### 2.2. Patients

Patients were consecutively included in the study if they: (1) fulfilled the criteria for sepsis/septic shock on admission to the ICU and (2) were ≥18 years of age [22]. The exclusion criteria were as follows: pregnancy, terminal illness with no chance for meaningful recovery, expected ICU length of stay of 24 h or less, or pre-existing KRT dependency.

The KDIGO AKI guidelines were used for AKI diagnosis [23]. Briefly, AKI stage 1: serum creatinine increase by ≥0.3 mg/dL (≥26.5 μmol/L) within 48 h or 1.5 to 1.9 times baseline which is known or presumed to have occurred within prior 7 days or urine output < 0.5 mL/kg/h for 6 to 12 h; AKI stage 2: serum creatinine 2.0 to 2.9 times baseline or urine output < 0.5 mL/kg/h for ≥12 h; AKI stage 3: serum creatinine 3.0 times baseline or increase in serum creatinine to ≥4.0 mg/dL (≥353.6 μmol/L) or initiation of kidney replacement therapy or in patients < 18 years a decrease in eGFR to < 35 mL/min per 1.73 m^2^ or urine output < 0.3 mL/kg/h for ≥24 h or anuria for ≥12 h. The serum creatinine concentration measured within 48 h prior to ICU admission was taken as the baseline serum creatinine level. These data are available in the hospital records of patients. Classification of AKI does not respond directly to the criteria of initiation of KRT. The inclusion criteria of KRT in our study met the KDIGO AKI guidelines which are hyperkalemia > 6 mmol/L, metabolic acidosis with pH < 7.2 and fluid overload > 20% in ventilated patients [23].

Patients were stratified according to the diagnosis of AKI and the need for KRT:

- **Group AKI(+)/KRT (+):** sepsis patients with AKI stage 3, who needed KRT to support kidney function;
- **Group AKI(+)/KRT(−):** sepsis patients with AKI stage 1–3, who did not need KRT to support kidney function;
- **Group AKI(−)/KRT(−):** sepsis patients without AKI and without the need for kidney function support;

The clinical status of the patients was determined with the Acute Physiology and Chronic Health Evaluation (APACHE) II score on admission to the ICU and Sequential Organ Failure Assessment (SOFA) score on admission and days 3, 5, and 7. The APACHE II score it is routinely used as a prediction tool for ICU patients and includes 12 physiological and 2 disease-related variables. The SOFA scale is commonly used in the ICU to monitor the severity of sepsis based on the status of the following systems: respiratory (PaO_2_/FiO_2_ index), cardiovascular (mean arterial pressure and the dose of vasopressors), hepatic (bilirubin concentration), coagulation (platelet count), kidney (creatinine concentration in blood/urine output), and neurological (Glasgow coma scale). All patients in the study were treated according to the Surviving Sepsis Campaign guidelines [24]. The results of routine laboratory tests were also included in the analysis.

Patients were followed up for 28 days after inclusion in the study.

### 2.3. Control Group

In order to compare the level of suPAR in cases with and without infection or renal failure, a control group was included in the study. This group consisted of adult patients admitted for first-time elective coronary artery bypass grafting under cardiopulmonary bypass. The exclusion criteria in the control group were as follows: the need for kidney function support either before or after surgery, infectious complications after surgery, poor myocardial function (ejection fraction < 40%), unstable angina, and other co-morbidities involving diabetes and renal or liver failure were excluded. In the control group, the level of suPAR was measured in blood samples collected in the operating room before the start of anesthesia.

### 2.4. Sample Collection and Measurement of the suPAR

Blood samples (2.7 mL, 3.2% sodium citrate as anticoagulant) were collected from patients on the day of admission to the ICU and on days 3, 5, and 7 of treatment. Plasma was immediately separated by centrifugation at 2000× *g* for 10 min, aliquoted, and stored at −70 °C for further analysis. In the control group, a blood sample (2.7 mL, 3.2% sodium citrate as anticoagulant) was collected only once at the operation theatre before anesthesia was induced. The quantitative determination of the soluble suPAR levels was performed with a suPARnostic enzyme-linked immunosorbent assay (ViroGates, Birkerød, Denmark). It is a commercially available diagnostic kit that has CE/IVD certification. All measurements were done in duplicate with appropriate controls.

### 2.5. Statistical Analysis

The data were analyzed with Statistica 13 (StatSoft, Inc., Tulsa, OK, USA). The distribution of the variables was not normal based on a Shapiro–Wilk test. Therefore, statistical analysis of the data was performed using non-parametric tests. Continuous variables were summarized with three statistics: mean, standard error, and minimum-maximum, while categorical variables were summarized as counts and fractions. The continuous variables between the study groups were compared with the Kruskal–Wallis ANOVA by ranks. In order to determine which groups were different from others, post hoc testing was conducted. Categorical variables were analyzed using a Pearson Chi-square test or Fisher exact test. Survival analysis of time to death was performed using the Kaplan–Meier curve and a Chi-square test. The predictive accuracy of the suPAR measurements on admission to the ICU was tested using receiver operating characteristic curve (ROC) analysis, by calculating the area under the curve (AUC), standard error (SE), and 95% confidence interval (CI). The Youden’s statistic was used to select the optimum suPAR cut-off point for KRT prognosis. Univariate and multivariate logistic regression analysis was performed to create a model predicting the need for KRT; the results were reported as odds ratio (OD) and 95% confidence intervals (CI). The choice of the best model was proposed based on the Akaike information criterion and the backward selection of the model. The statistical analysis was conducted using R 3.6.01: R Core Team (2013). Statistical significance was determined as *p* ≤ 0.05.

## 3. Results

In the 10-month study period, 51 patients met the inclusion/exclusion criteria and were included in the analysis. The majority (82%) had septic shock diagnosed on admission to the ICU and 18% had sepsis. The mean APACHEII score for the entire group was 22 points, the mean SOFA score was 12 points, and 96% of patients required mechanical ventilation on ICU admission. In most cases, the source of sepsis was infection of the abdominal cavity (*N* = 25) or lungs (*N* = 19); in addition, there was urinary tract infection in 3 cases, and of soft tissue in 4. The ICU mortality was 39%.

Based on the need for renal function support and a diagnosis of AKI on admission to the ICU, patients were placed into group AKI(+)/KRT(+), (*N* = 18; 35%), group AKI(+)/KRT(−), (*N* = 21; 41%), or group AKI(−)/KRT(−), (*N* = 12; 24%). The diagnosis of AKI and the need for KRT were then confirmed/ruled out for each patient on days 3, 5, and 7 of the study. The study groups did not differ in terms of age (*p* = 0.239) and gender (*p* = 0.403). The levels of systemic inflammatory response markers, such as CRP, WBC, and procalcitonin, were elevated in the patients, but did not differ significantly between the study groups. The baseline characteristics of the study groups are presented in Table 1.

**Figure 1:**
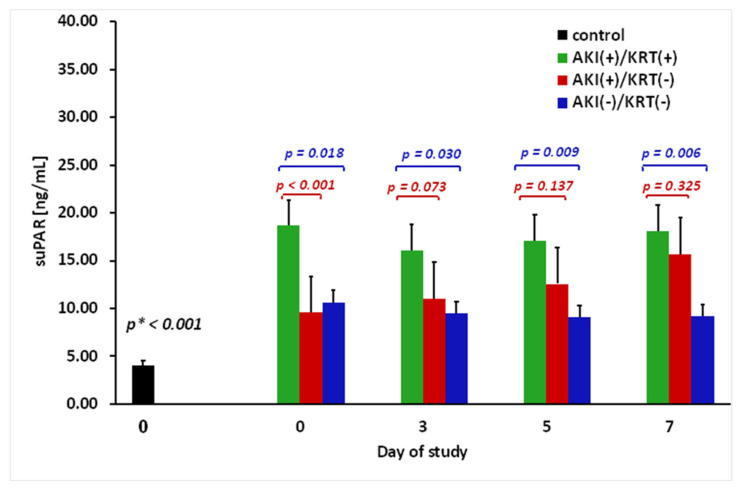
Plasma levels of the suPAR measured on the 0, 3rd, 5th, and 7th day of the study. The *p*-value represents a significant difference in the suPAR level between the study groups: AKI(+)/KRT(+) and AKI(+)/KRT(−) and between AKI(+)/KRT(+) and AKI(−)/KRT(−) calculated on each day of observation using the Kruskal–Wallis test with post hoc analysis. The difference between groups AKI(+)/KRT(−) and AKI(−)/KRT(−) was not significant on any of the observation days. The p* value represents the difference in the suPAR level between the control and study group (day 0).

Additionally, blood samples were taken from 20 patients admitted for elective cardiac surgery (control group) to test suPAR levels in cases without infection or renal failure and to compare them with the results of patients with sepsis (the analysis is presented in Figure 1). The mean age in the control group was 66 ± 2 years (38–84 years), and males accounted for 60%. The study group and the control group did not differ in terms of age (*p* = 0.494) and gender (*p* = 0.591). After surgery, none of the patients in the control group developed renal failure or infectious complications.

### 3.1. The suPAR Level with an AKI Diagnosis and the Need for KRT

The baseline level of suPAR in all sepsis patients enrolled in the study was significantly higher compared to the control group (13.01 ± 1.24 ng/mL (3.28–39.11) vs. 4.05 ± 0.46 ng/mL (1.45–8.85), *p* < 0.001). Further analysis of the suPAR levels between the three study groups using the Kruskal–Wallis test showed significant differences with p values of 0.001, 0.050, 0.029, and 0.030 on days 0, 3, 5, and 7, respectively. The highest suPAR levels were reported in patients with AKI who required KRT to support renal function (Figure 1, green bars). However, the results of the post hoc analysis revealed that among AKI patients, the difference between the AKI(+)/KRT(+) and AKI(+)/KRT(−) groups was statistically significant on ICU admission (day 0, *p* < 0.001) but not on days 3, 5, and 7. Differences in the suPAR levels between the AKI(+)/KRT(+) and AKI(−)/KRT(−) groups were significant on all study days, and at the same time points there were no differences in the suPAR level between the AKI(+)/KRT(−) and AKI(−)/KRT(−) groups. Figure 1 illustrates the comparison for all patients subdivided according to the need for renal function support and a diagnosis of AKI.

### 3.2. Survival Analysis

**Figure 2:**
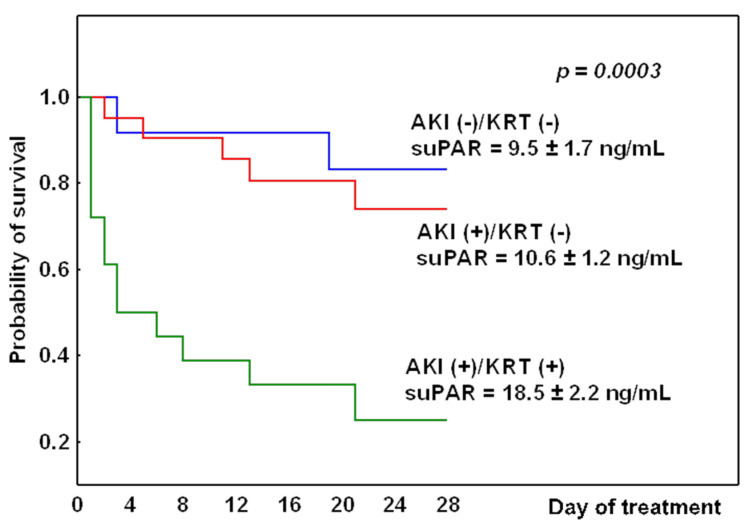
Comparison of 28-day survival in the three studied groups (*p* = 0.0003) based on the AKI diagnosis and the requirement for KRT on admission to the ICU.

The analysis of survival was performed based on the AKI diagnosis and the requirement for renal function support on admission to the ICU: AKI(+)/KRT(+), AKI(+)/KRT(−) or AKI(−)/KRT(−). Figure 2 illustrates the Kaplan–Meier curves for 28-day survival in the three study groups. Patients in the AKI(+)/KRT(+) group were characterized by the highest level of the suPAR and, at the same time, had the lowest probability for 28-day survival (*p* = 0.0003).

### 3.3. The suPAR as an KRT Prediction Tool

The suPAR had the ability to predict the need for KRT in patients with sepsis on admission to the ICU with an AUC of 0.801 (95% CI 0.676–0.925, *p* < 0.001). The optimal cut-off value for the baseline suPAR level was 10.422 ng/mL, with sensitivity of 88.9% and specificity of 63.6%. The results of the receiver operating characteristic analysis and the Youden’s statistic are shown in Table 2.

In patients with the suPAR concentration above the cut-off value, the requirement for KRT was significantly higher (89%, *p* < 0.001). Moreover, patients diagnosed with AKI and with the suPAR concentration above the cut-off value had significantly higher 28-day mortality (65%, *p* = 0.005).

In addition, a univariate and multivariate logistic regression analysis was performed to create a model predicting the need for KRT. The choice of the variables from the set of biomarkers (suPAR, creatinine, urea, pH, potassium, PCT, and lactate) and covariates (age, SOFA, APACHE II, AKI, shock) was determined by the minimizing of the Akaike information criterion and the backward selection of the model. Therefore, the only variables that were included in the final model were the suPAR, creatinine, urea, APACHEII, and SOFA. The initial suPAR and APACHEII were significant predictors of KRT. The initial creatinine, urea, and SOFA score had no statistical significance in the model. Results of analysis are presented in Table 3.

### 3.4. The Effect of Surgery on suPAR

In order to distinguish the effect of surgery on the suPAR level, analyses were performed in two subgroups, surgical and medical. The first subgroup consisted of postoperative patients with abdominal cavity infection as a source of sepsis (*N* = 25) and the second subgroup consisted of patients with lung, urinary tract, and soft tissue infections as a source of sepsis (*N* = 26). In the surgical subgroup, the suPAR level was 12.08 ± 1.67, 12.88 ± 2.09, 14.59 ± 2.25, 15.27 ± 3.11 ng/mL, and in the medical subgroup the suPAR level was 13.91 ± 1.84, 12.71 ± 1.84, 12.08 ± 1.51, and 13.34 ± 2.01 ng/mL on days 0, 3, 5, and 7, respectively, and there were no differences in the suPAR levels between the two subgroups throughout the observation period (*p* > 0.05).

## 4. Discussion

The results of this preliminary study indicate that sepsis patients have significantly increased blood levels of the suPAR, with the highest concentrations found in those who developed AKI and required KRT to support renal function. The optimal suPAR cut-off point for predicting the need for KRT was established at 10.422 ng/mL with an AUC of 0.801 (sensitivity 0.889, specificity 0.636). Moreover, patients with AKI requiring KRT had the lowest probability of survival compared to patients with AKI without KRT and patients without AKI. In our previous study of patients undergoing cardiac surgery under cardiopulmonary bypass, it was shown that surgical stress and immune activation had no effect on the suPAR level: there was a significant increase in pro-inflammatory cytokines and CRP after surgery, reflecting the activation of the systemic inflammatory response induced by surgery, but no change in suPAR levels was observed over the same period [25]. In the present study, a control group of patients undergoing cardiac surgery was enrolled to demonstrate differences in the suPAR levels in sepsis and non-sepsis cases, regardless of the diagnosis of AKI.

The suPAR is a soluble form of the membrane bound urokinase-type plasminogen activator receptor (uPAR), a glycosyl-phosphatidylinositol-anchored (GPI) three-domain receptor protein, expressed in a variety of cells, including podocytes, immune cells, and endothelial cells [2]. Changes in the suPAR level in the circulation reflect the aggregate activity of the uPAR system with respect to innate immune activity, proteolysis, and extracellular matrix remodeling [1]. Attention was also paid to the role of the plasminogen activator-1 (PAI-1), urokinase plasminogen activator (uPA) and the uPAR on the surface of podocytes, which additionally binds integrin β1 and causes podocyte detachment. The soluble (suPAR) as well as the membrane-bound form (uPAR) of the urokinase receptor activates beta-3 integrin (β3) in podocytes. The β3 integrin is one of the main proteins anchoring podocytes in the basement membrane of renal glomerules and increased activation impairs the functions of the foot processes of the podocytes, changes their shape, and consequently damages the filtration membrane, which can lead to albuminuria and glomerulonephritis [26]. The results of the experimental study also showed that the suPAR increases the energy demand of cells and induces oxidative stress in tubular cells, which are prone to ischemia-reperfusion damage [13,27]. High levels of suPAR have been shown to promote tubular fibrosis in an integrin dependent manner [28]. An experimental model has shown that these deleterious changes in podocytes and tubular cells can be prevented using anti-uPAR antibodies [29]. The methods suggested in the literature to protect podocytes from lesions may include: blocking the suPAR by specific antibodies, using a β3 integrin inhibitor or antibodies against the β3 integrin, and inhibiting the interactions between the suPAR and the β3 integrin by eliminating the suPAR during plasmapheresis [26].

Currently, the assessment of kidney function in critically ill patients is based on clinical judgment and conventional criteria: measuring the parameters indirectly indicative of glomerular filtration disturbances, such as serum creatinine, urea concentration, and urine output. Changes in serum creatinine and urea concentration are not always representative of true kidney damage and are, therefore, ineffective in predicting outcome in AKI patients. In recent years, there has been some progress in risk stratification, prevention, and treatment of AKI and the use of the suPAR and other markers of organ damage such as tissue neutrophilic gelatinase-associated lipocalin (NGAL), cystatin C, kidney injury molecule 1 (KIM-1), interleukin 18 (IL 18), inhibitors of metalloproteinase-2 (TIMP-2), and insulin-like growth factor -7 binding protein (IGFBP-7) offer hope for the development of an algorithm to predict the need for KRT in patients with AKI [21,30,31,32]. In a study by Liu et al., TIMP-2 and IGFBP-7 were identified as potential predictors of AKI [33]. TIMP-2 and IGFBP7 are markers of G1 cell cycle arrest and the levels of these proteins increased during the early period after renal tubular cell injury [34]. The combination of these two biomarkers showed good diagnostic accuracy in predicting AKI with an AUC of 0.86 (sensitivity 0.83, specificity 0.72). In another study, a panel of potential biomarkers was tested for risk stratification in patients with septic AKI requiring KRT; the baseline suPAR values predicted KRT demand with good diagnostic accuracy, and the optimal cut-off point for the suPAR level was 8.53 ng/mL with an AUC of 0.83 (sensitivity 84.2, specificity 82.7) [21].

It has previously been found that in patients with normal kidney function at baseline, high levels of suPAR may be associated with the risk of developing chronic kidney disease; the risk of progression to chronic kidney disease in patients with suPAR levels > 4.02 ng/mL (the highest quartile) was 3.13 times higher than the risk in patients with suPAR < 2.37 ng/mL (the lowest quartile) in the population of patients undergoing cardiac catheterization [11]. Recently, Hayek et al. investigated whether high levels of suPAR predispose to the development of AKI in patients undergoing coronary angiography and cardiac surgery [13]. They found that among those who required ICU treatment, the incidence of acute kidney injury was as high as 53% in the highest suPAR quartile (≥9.44 ng/mL), which is close to the cut-off value established in our study: 10.422 ng/mL, with AUC 0.801, sensitivity 88.9%, and specificity 63.6%. When we conducted our study, there were no SARS-CoV-2 infected patients in the ICU, therefore we have no own experience as to whether suPAR is associated with the development of AKI in patients with COVID-19 infection. A recently published results of the ISIC study (International Study of Inflammation in COVID-19) indicate that suPAR levels > 6.86 ng/mL were associated with a 9.15-fold increase in the likelihood of developing AKI and a 22.86-fold increase in the likelihood of requiring dialysis [35]. The cut-off point for suPAR > 6.86 ng/mL is lower than that calculated in our study, but the ISIC study included the general population of patients hospitalized for COVID-19, while our study included patients with sepsis and septic shock.

The optimal timing to start KRT in critically ill patients with AKI is still under debate and has been the subject of several clinical trials assessing the role of biomarkers in decision making. The ELAIN and STARRT-AKI clinical studies investigated the usefulness of NGAL in patients with AKI [36,37]. In the ELAIN study, a fixed NGAL threshold was used as an inclusion criterion, defining the need for early KRT as AKI KDIGO stage 2 and plasma NGAL > 150 ng/mL and delayed KRT as AKI stage 3. The study found that NGAL indicated patients with progressive AKI, and the early start of KRT significantly reduced the 90-day mortality compared to a delayed start (39.3% vs. 54.7%). In the STARRT-AKI study, the NGAL level ≥ 400 ng/mL, along with a twofold increase in serum creatinine and oliguria, was used to guide the early start of KRT; the results were not as expected, because the early initiation of KRT in patients with NGAL levels ≥ 400 ng /mL had no effect on mortality. According to the KDIGO MTC guidelines, KRT should be started when life-threatening changes in the fluid, electrolyte and acid-base balance occur, and it is recommended to take into account the wider clinical context and trends in laboratory results when deciding to initiate KRT [38]. A recently published meta-analysis showed that the early start of KRT was not associated with an increase in survival in critically ill patients with AKI. Moreover, an early start of KRT may lead to unnecessary exposure to KRT in some patients, resulting in a higher incidence of KRT-associated adverse events, the increased use of ICU resources, and the correspondingly higher nursing workload. Perhaps only critically ill patients with specific clinical indication, such as severe acidosis, pulmonary edema, and hyperkalemia, may benefit from the early initiation of KRT [39]. Our study was designed to be observational; KRT in patients with AKI was initiated according to the KDIGO AKI guidelines and the beneficial effects of an early vs. late start of KRT were not compared. It should be emphasized that patients in the AKI(+)/KRT(+) group were characterized by the highest level of the suPAR, and at the same time they had lowest 28-day survival probability. In this group, the initial suPAR concentration of 18.52 ± 2.2 ng/mL was associated with 72% mortality, despite the implementation of KRT. It is possible that starting KRT earlier in this group would have improved the outcome.

Another very important problem in evaluating the further usefulness of biomarkers for predicting the need for KRT in patients with AKI is the time point of the evaluation. In a study by Nusshag et al. the combination of TIMP-2 × IGFBP-7 levels after 24 h of the suPAR levels at baseline were the strongest predictors of septic AKI courses requiring KRT [21]. In another study that investigated groups with early versus standard initiation of KRT (furosemide stress test trial), the plasma levels of NGAL were elevated in both groups at the time of randomization; however, there were no significant differences in NGAL either within the treatment groups or between groups at any time of observation [40]. In a study by Koch et al. the suPAR was identified as a stable marker for predicting disease severity and the risk of death in ICU patients. A high suPAR level on admission (>8 ng/mL) and on day 3 (>13 ng/mL) were independent predictors of both ICU and long-term mortality [41]. In our study, the highest suPAR concentrations were in the AKI(+)/KRT(+) group throughout the observation period. The suPAR level had the best ability to predict the need for KRT on admission to the ICU with an AUC of 0.801 and with an optimal cut-off point of 10.422 ng/mL. Moreover, according to the multivariate logistic regression analysis, the best model predicting the need for kidney replacement therapy in patients with sepsis included initial suPAR and APACHE II score: an elevated suPAR and APACHE II indicated a significantly higher risk of the need for kidney replacement therapy.

The possible role of suPAR in the immune response remains to be investigated further. It is worth noting that suPAR has been extensively studied in the sepsis patient population, but elevated levels of this biomarker have also been shown to be of prognostic value in other diseases, including HIV-1 infection, tuberculosis, meningitis, as well as various forms of cancer [7,42,43,44,45]. The results of these studies indicate that measuring the concentration of suPAR may be useful for the clinical management of various diseases.

Our study has several limitations. It is a single-center study and the relevance of the findings is limited by the small sample size. Therefore, the diagnostic threshold for the suPAR and trends presented, however similar to the results obtained in earlier studies, should be further validated. The clinical status of patients was severe as indicated by the high APACHE II and SOFA score, and the majority of patients had septic shock diagnosed on admission to the ICU. The APACHE II and SOFA scores and the ratio of AKI patients were higher than in previous reports [46,47]. All these factors had an impact on the high mortality rate. It should also be emphasized that the function of the suPAR and the regulatory mechanisms of its action in critically ill are not well understood. The changes in suPAR values observed in subsequent days could also have been influenced by numerous additional stress factors that appeared during the development and intensification of the disease.

## 5. Conclusions

There is an unmet clinical need for the early diagnosis of AKI. The highest concentrations of suPAR were found in patients who developed AKI and required KRT; therefore, suPAR measurements may constitute an important element of diagnostics and therapy in this group of critically ill patients. Measuring an additional marker would increase the cost of treatment, but the potential benefit to the patient would justify that increase. More targeted validation studies are needed to evaluate the results with different suPAR thresholds. The results of our study and previous studies on the usefulness of kidney injury biomarkers in predicting the need for KRT have important scientific and clinical implications; however, the clinical decision to initiate KRT is not simply based on the severity of the organ damage, but rather on an imbalance between the remaining kidney function and factors such as disease severity, comorbidities, and fluid overload.

## Figures and Tables

**Table 1:** Baseline characteristics of patients with sepsis. Patients were divided according to the need for kidney replacement therapy (KRT +/−) and a diagnosis of acute kidney injury (AKI +/−) on admission to the ICU. The KDIGO AKI guidelines were used for AKI diagnosis.

Variable	AKI(+)/KRT(+)	AKI(+)/KRT(−)	AKI(−)/KRT(−)	*p*
	(*N* = 18)	(*N* = 21)	(*N* = 12)	
Age [years]	70 ± 2 (47–85)	69 ± 2 (41–92)	60 ± 52 (19–88)	0.239
Male *N* [%]	10 (56)	9 (43)	8 (67)	0.403
APACHE II score	27 ± 2 (13–38)	19 ± 1(9–31)	18 ± 1 (9–25)	0.001
SOFA score	14 ± 1 (6–18)	10 ± 1 (4–16)	10 ± 2 (2–16)	0.005
Septic shock/sepsis [*N*]	17/1	19/2	6/6	0.003
Mechanical ventilation [*N*]	17/1	20/1	12/0	0.736
Diagnosis on admission [*N*]				0.103
Intra-abdominal infection	8	13	4	
Pneumonia	5	6	8	
UTI	3	0	0	
Skin, soft tissue infection	2	2	0	
Procalcitonin [ng/mL]	107 ± 67 (0.7–1127)	20 ± 10 (0.5–234)	14 ± 7 (0.2–89)	0.175
WBC [10^3^/µL]	16 ± 6 (0.02–47)	14 ± 1 (2–32)	18 ± 1 (13–29)	0.227
CRP [mg/L]	180 ± 41 (7–498)	218 ± 27 (6–472)	264 ± 52 (25–552)	0.380
Creatinine [mg/dL]	3.0 ± 0.5 (0.9–6.9)	1.9 ± 0.2 (0.7–5.9)	1.1 ± 0.3 (0.5–5.4)	<0.001
Urea [mg/dL]	95 ± 9 (29–169)	84 ± 9 (29–169)	53 ± 7 (31–111)	0.048
Lactate [mmol/L]	10.3 ± 1.7(1.5–26.0)	3.0 ± 0.4 (0.8–10.2)	2.5 ± 0.8 (0.6–11.4)	0.035
ICU stay [days]	7 ± 2 (2–36)	9 ± 1 (2–219)	15 ± 3 (3–37)	0.007
28-day mortality *N* [%]	13 (72)	5 (24)	2 (17)	0.001

Data are presented as means ± standard error (minimum-maximum) or counts and fractions. *p* values illustrate comparisons between the three study groups (ANOVA Kruskal–Wallis or Chi-square test). APACHE, Acute Physiology and Chronic Health Evaluation; SOFA, Sequential Organ Failure Assessment; UTI, urinary tract infection; CRP, C-reactive protein; WBC, white blood cell count; ICU, intensive care unit.

**Table 2:** The receiver operating characteristic analysis for predicting kidney replacement therapy (KRT) based on the suPAR level.

All Patients	KRT	AUC	*p*	Cut-Off	Sensitivity	Specificity
(*N*)	(*N*)	(95% CI)		[ng/mL]	(95% CI)	(95% CI)
51	18	0.801	<0.001	10.422	0.889	0.636
		(0.676–0.925)			(0.639–0.980)	(0.451–0.790)

suPAR: soluble urokinase-type plasminogen activator receptor; AUC, area under the curve; CI, confidence interval.

**Table 3:** Results of a multivariate logistic regression analysis model predicting the need for kidney replacement therapy in patients with sepsis.

	Univariate Analysis			Multivariate Analysis		
	Odds Ratio	95% CI	*p*	Odds Ratio	95% CI	*p*
suPAR	1.14	1.05–1.27	0.004	1.16	1.04–1.32	0.009
APACHE II	1.22	1.02–1.40	<0.001	1.18	1.02–1.45	0.044
SOFA	1.43	1.16–1.85	0.002	1.32	1.00–1.91	0.080
creatinine	1.69	1.63–2.66	0.011	2.02	1.02–5.48	0.099
urea	1.01	0.99–1.02	0.106	0.79	0.94–1.00	0.182
shock	5.44	0.87–105.71	0.125			
pH	5.78 × 10^−6^	1.23 × 10^−9^–3.02 × 10^−3^	0.001			
potassium	2.24	1.14–5.01	0.028			
PCT	1.06	0.99–1.01	0.198			
lactate	1.34	1.16–1.64	<0.001			
age	1.02	0.98–1.08	0.258			

CI: confidence interval; suPAR: soluble urokinase-type plasminogen activator receptor; APACHE II: Acute Physiology and Chronic Health Evaluation score II; SOFA: Sequential Organ Failure Assessment score; PCT: procalcitonin.

## Data Availability

The data that support the findings of this study are available on request from the corresponding author.
